# A brief experience of blindness

**Published:** 2008-12

**Authors:** Hessom Razavi

**Affiliations:** Senior House Officer in Ophthalmology, London House, Mecklenburgh Square, London WC1N 2AB, UK. Email: hessom.razavi@gmail.com

**Figure:**
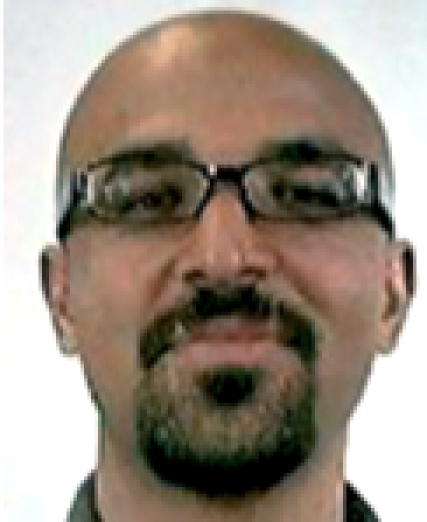


Some months ago, I observed a blind pedestrian negotiating a busy London street with remarkable speed and confidence. “Just how does he do it?”, I thought to myself. I decided that I, as an eye health professional, could benefit from a brief experience of what it was like to be ‘blind’. This became the inspiration for a fundraising event, which involved me having my eyes covered for 24 hours, and being effectively ‘blind’, under close supervision from a sighted guide.

Living with sudden and complete visual loss is a high-risk endeavour, and ‘pretending’ to lose one's sight is no less risky. For this reason, prior to the event, my sighted guide, Puneet Sayal, and I underwent intensive visual awareness training. This involved learning a range of safe coping techniques, both indoors and out, such as opening doors, climbing stairs, and pouring hot drinks. Even the simplest activity could be challenging.

On the day of the event, with close guidance from my guide, I went about my normal daily routine: getting dressed, preparing and eating breakfast, walking to university, attending classes, etc.

I quickly noticed that some people treated me differently now that my eyes were covered: they were either more or less attentive than usual, and they sometimes addressed me indirectly by talking to my guide. Some seemed disturbed by my condition, others overeager to help.

I found I was less confident, found it more difficult to concentrate in class, and was not able to contribute as much as usual. I was completely dependent on my guide and experienced a severe loss of freedom. As the day progressed, I began to feel stressed and became anxious to remove my eye coverings. When they finally came off after 24 hours, the light was actually painful. I also felt quite emotional and remained unsettled for a few hours.

## Reflections after the event

The experience was deeply unsettling, but very valuable to me. Thanks to my guide, I never felt unsafe. I was more disturbed by my diminished confidence, independence, and ability to contribute. I cannot suggest that I now ‘understand’ what it is like to be blind, but I did learn the following lessons:

- Blind people must be treated normally—inclusion and dignity are paramount
- Efforts to help blind people can both help and hinder them
- Ask first before offering help and be precise with instructions
- Look at blind people and speak to them directly, using normal language
- Leaving a blind person without announcing it is unkind and embarrassing
- Ask how and at what pace a blind person would like to be guided
- Make sure the person you are guiding is safe, but stay relaxed, and remember not to pull, but to walk with him/her holding your arm
- Being blind is a constant challenge
- Rehabilitation training is crucial.

